# Macrofaunal assemblages associated with the sponge *Sarcotragus foetidus* Schmidt, 1862 (Porifera: Demospongiae) at the coasts of Cyprus and Greece

**DOI:** 10.3897/BDJ.4.e8210

**Published:** 2016-05-30

**Authors:** Christina Pavloudi, Magdalini Christodoulou, Michalis Mavidis

**Affiliations:** ‡ Institute of Marine Biology, Biotechnology and Aquaculture, Hellenic Centre for Marine Research, Heraklion, Crete, Greece; § Department of Biology, University of Ghent, Ghent, Belgium, Department of Microbial Ecophysiology, University of Bremen, Bremen, Germany; | Department of Zoology, School of Biology, Aristotle University of Thessaloniki, Thessaloniki, Greece

**Keywords:** *Sarcotragus foetidus*, Porifera, Demospongiae, macrofauna, Greece, North Aegean Sea, Cyprus, Levantine Basin, Eastern Mediterranean

## Abstract

**Background:**

This paper describes a dataset of macrofaunal organisms associated with the sponge *Sarcotragus foetidus* Schmidt, 1862, collected by scuba diving from two sampling sites: one in Greece (North Aegean Sea) and one in Cyprus (Levantine Sea).

**New information:**

This dataset includes macrofaunal taxa inhabiting the demosponge *Sarcotragus foetidus* and contributes to the ongoing efforts of the Ocean Biogeographic Information System (OBIS) which aims at filling the gaps in our current knowledge of the world's oceans. This is the first paper, to our knowledge, where the macrofauna associated with *S. foetidus* from the Levantine Basin is being recorded.

In total, 90 taxa were recorded, from which 83 were identified to the species level. Eight of these species are new records for the Levantine Basin. The dataset contains 213 occurrence records, fully annotated with all required metadata.

It is accessible at http://lifewww-00.her.hcmr.gr:8080/medobis/resource.do?r=organismic_assemblages_sarcotragus_foetidus_cyprus_greece

## Introduction

It is well known that sponges host a variety of macrobenthic organisms, providing them with shelter and constant food supply (Fishelson 1966, Koukouras et al. 1985, Koukouras et al. 1996, Koukouras et al. 1992, Çinar and Ergen 1998, Ilan et al. 1994). The relationship between sponges and their associated macrofaunal species has been investigated by several scientists (e.g. Pearse 1932, Pearse 1950, Arndt 1933, Bacescu 1971, Frith 1976, Long 1968).

**Figure 1.**
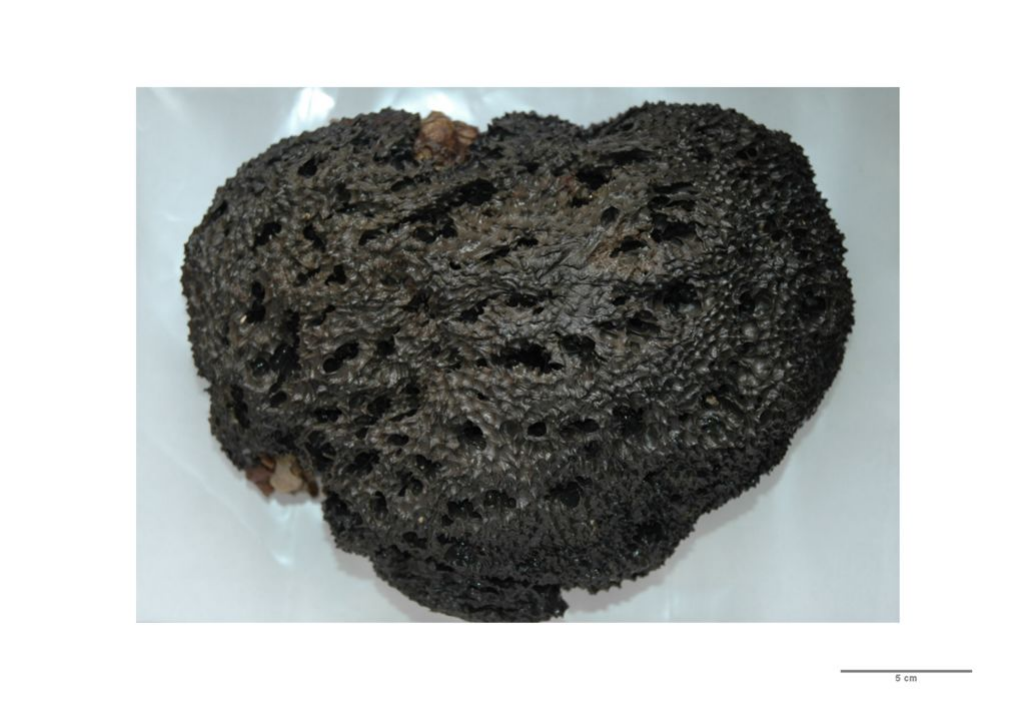
Photo of the demosponge *Sarcotragus foetidus* Schmidt, 1862, collected in the course of the study.

The sponge *Sarcotragus foetidus* Schmidt, 1862 (Fig. 1) belongs to the class of Demospongiae and, more specifically, to the subclass of Keratosa, i.e. sponges with skeleton comprised of spongin fibers (order Dictyoceratida, family Irciniidae).

This particular sponge species has an extensive network of small and large channels and cavities, and thus allows a variety of benthic invertebrates to inhabit them. Its surface is characterized by conules of 2–3 mm height, which are 10–15 mm apart from one another. The main skeleton is composed by a reticulate network of primary (ca. 100–200 µm in diameter) and secondary (ca. 50–100 µm in diameter) fibres (Manconi et al. 2013). Interestingly, this species was even mentioned by Aristotle, who had named it "Aplysias", meaning that it cannot be cleaned and used as a bath sponge, although its external morphology resembles to that of common bath sponges (Voultsiadou and Vafidis 2007).

The macrofaunal assemblages associated with *S. foetidus* have been investigated by several authors (e.g. Çinar and Ergen 1998, Çinar et al. 2002, Koukouras et al. 1985, Pansini and Daglio 1981, Rützler 1976) and in different study sites (e.g. Aegean Sea, Ligurian Sea, Tunisian coasts), although they have not been studied yet in the Levantine Sea.

## General description

### Purpose

This dataset includes species found associated with the demosponge *S. foetidus*. The sample sponges were collected from Greece (Linaraki, Sithonia, Halkidiki) and Cyprus (Milouria, Kissonerga, Pafos). Sampling in Cyprus was conducted in January of 2003 and August of 2003 and 2007, at depths between 5 and 10 meters. Sampling in Greece was conducted in February and July of 2003, in depths between 14 and 17 meters.

## Project description

### Personnel

Christina Pavloudi, HCMR (sample collection, taxonomic identification, data management), Michalis Mavidis, Aristotle University of Thessaloniki (sample collection, taxonomic identification), Magdalini Christodoulou, Aristotle University of Thessaloniki (sample collection, taxonomic identification), Athanasios Koukouras, Aristotle University of Thessaloniki (sample collection).

### Study area description

**Figure 2.**
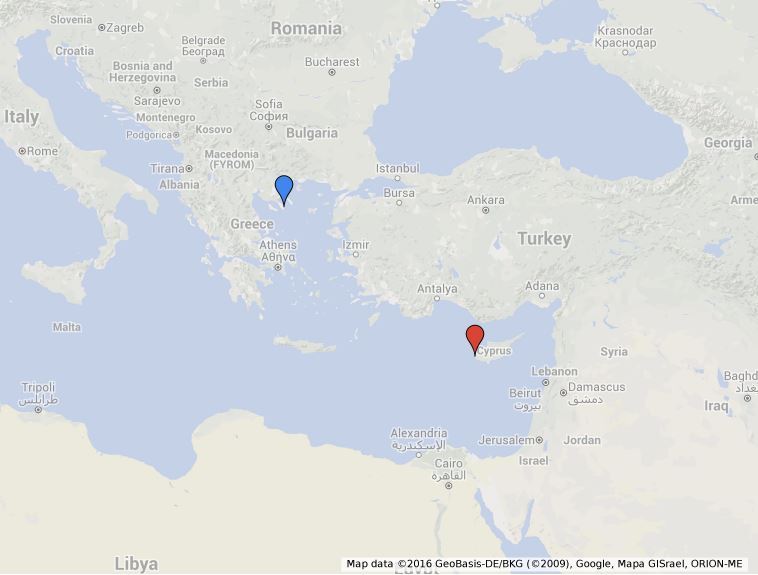
Geographical location of the study area indicating the sampling stations.

Samples were collected from one location at the coast of Halkidiki (Greece) and one location at the coast of Cyprus (Fig. 2). The two study sites can be distinguished based on their trophic state index. North Aegean Sea can be characterized as mesotrophic to eutrophic (Kyriakidis et al. 2015), in contrast to the oligotrophic Levantine Basin (Duineveld et al. 2000). Both study sites are rocky shores dominated by different species of photohilic algae, thus no obvious differences in the sponge associated fauna can be attributed to differences in the substrate type.

**Linaraki**: The sampling site is located on the peninsula of Sithonia, in the North Aegean Sea (Fig. 3a). It is a moderately exposed rocky shore with a photophilic algal assemblage dominated by *Ellisolandia elongata*.

**Milouria**: The sampling site is located close to the town of Pafos (Southwest Cyprus) (Fig. 3b. It is an exposed rocky shore with a photophilic algal assemblage dominated by the the non-indigenous species *Palisada perforata*.

## Sampling methods

### Study extent

Samples were collected at single time points. Three sponges were collected in the winter and two in the summer season from Greece. In addition, three sponges were collected in the winter and four sponges (one in 2003 and three in 2007) in the summer season from Cyprus.

### Sampling description

Samples were collected by scuba divers. Each sponge was first covered with a plastic bag and was subsequently detached from the substrate, manually, with a knife. Once ashore, formalin was added in every sample to a final concentration of 5% and the samples were stored in jars.

Upon return to the laboratory, the epifauna of each sponge was collected initially. The formalin solution contained in the plastic bags was filtered through a 0.5 mm mesh size sieve in order to collect epifaunal organisms that were detached from surface of the sponges. Then, the surface and volume of each sponge was measured. Sponge volume was measured by water displacement. Afterwards, sponges were cut in smaller pieces and the animals found in the sponge channels were collected.

### Quality control

All scientific names were standardised against the World Register of Marine species using the Taxon Match tool. Taxon names were also kept in the dataset as they had been originally recorded, with a reference to the currently accepted name.

## Geographic coverage

### Description

Includes one location in Cyprus (Milouria, Kissonerga, Pafos) and one location in Greece (Linaraki, Sithonia, Halkidiki) (Christodoulou et al. 2013). More information can be found in Table 1.

### Coordinates

34.43 and 40.44 Latitude; 23.58 and 32.85 Longitude.

## Taxonomic coverage

### Description

The dataset comprises distribution information for 90 taxa, belonging in 48 families and 8 phyla. Detailed information is presented in Table 2. Of these, 8 species have been recorded for the first time in the Levantine Basin.

**Figure 4.**
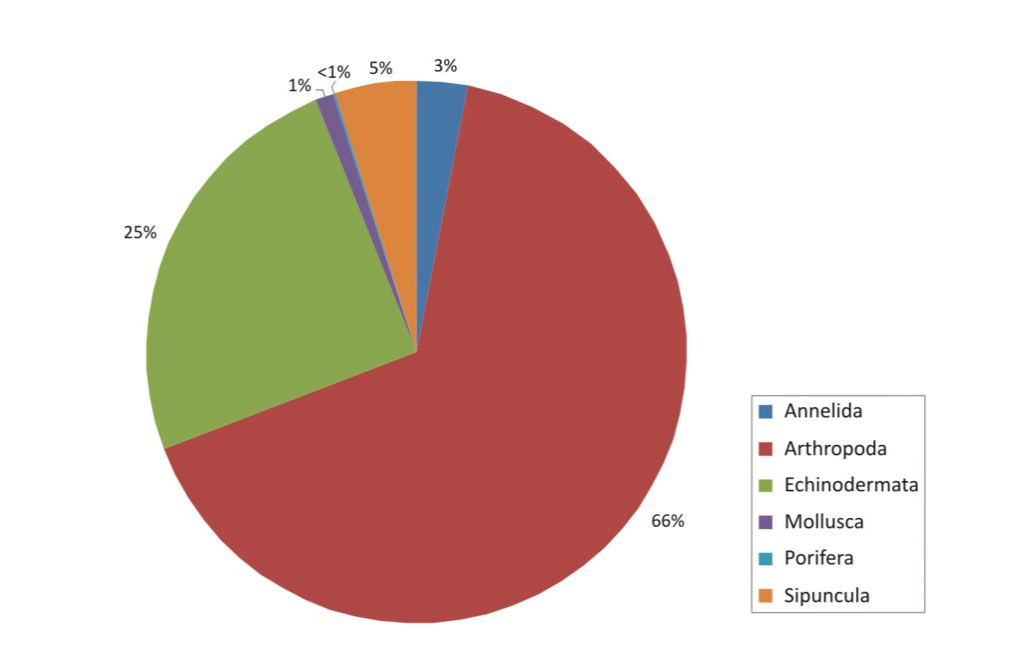
Percentages of main phyla in the summer samples of Cyprus, as calculated based on the individual count of species per total sponge volume.

**Figure 5.**
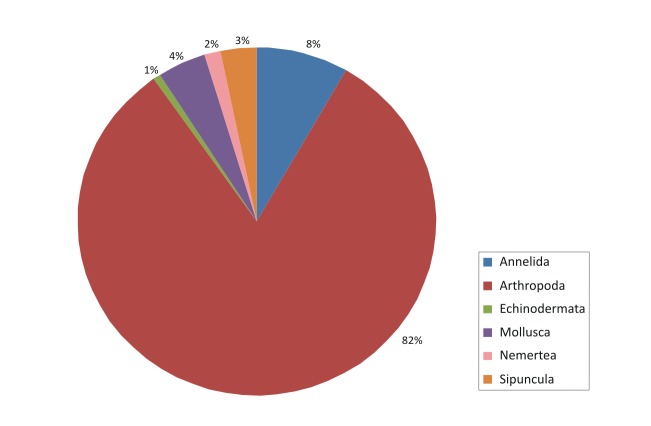
Percentages of main phyla in the winter samples of Cyprus, as calculated based on the individual count of species per total sponge volume.

**Figure 6.**
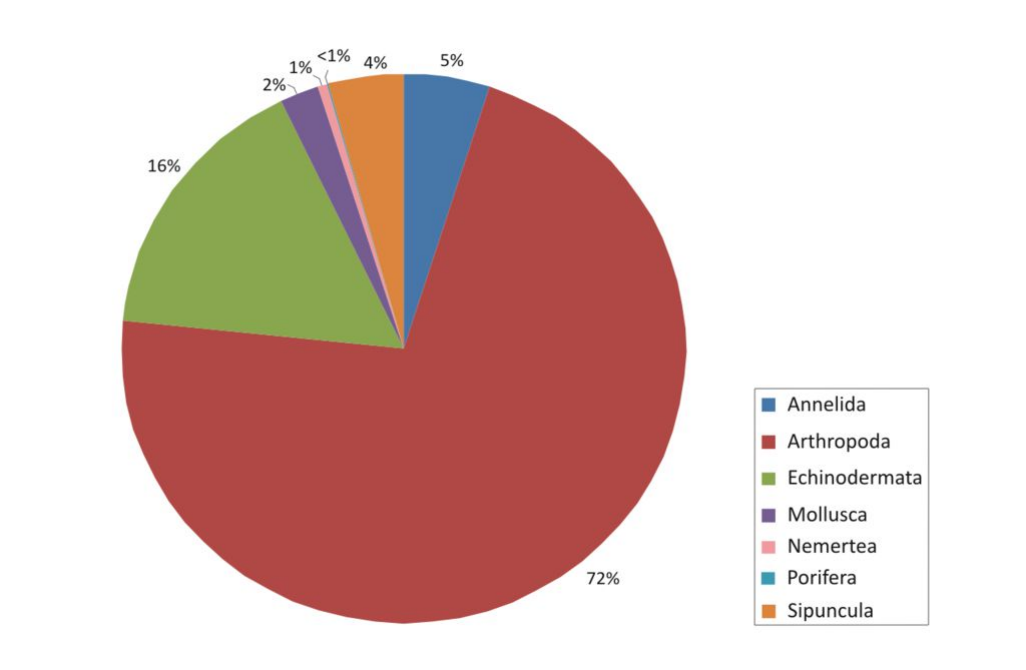
Percentages of main phyla in all the samples of Cyprus, as calculated based on the individual count of species per total sponge volume.

**Figure 7.**
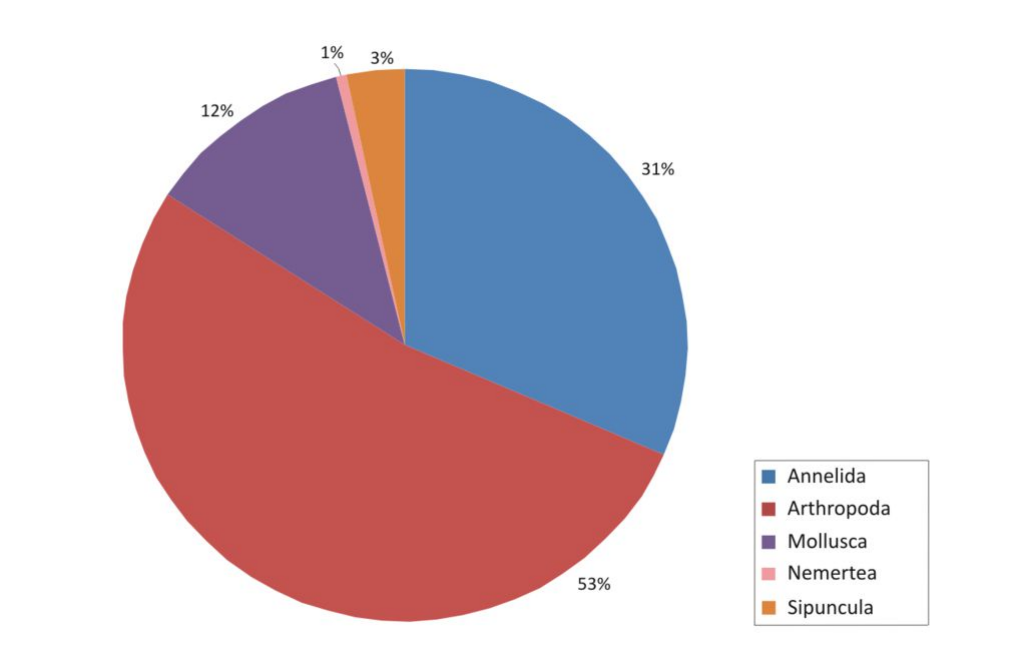
Percentages of main phyla in the summer samples of Greece, as calculated based on the individual count of species per total sponge volume.

**Figure 8.**
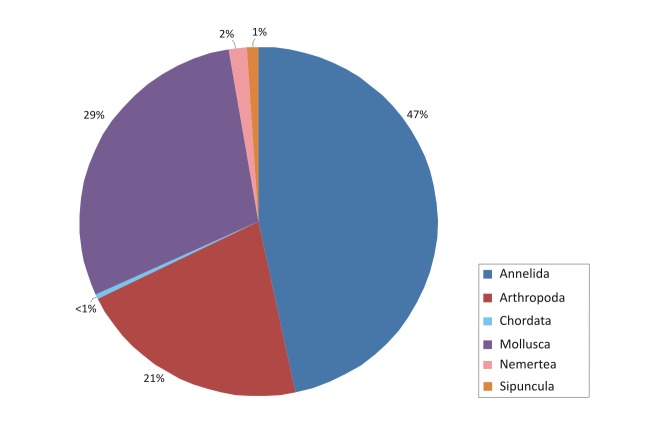
Percentages of main phyla in the winter samples of Greece, as calculated based on the individual count of species per total sponge volume.

**Figure 9.**
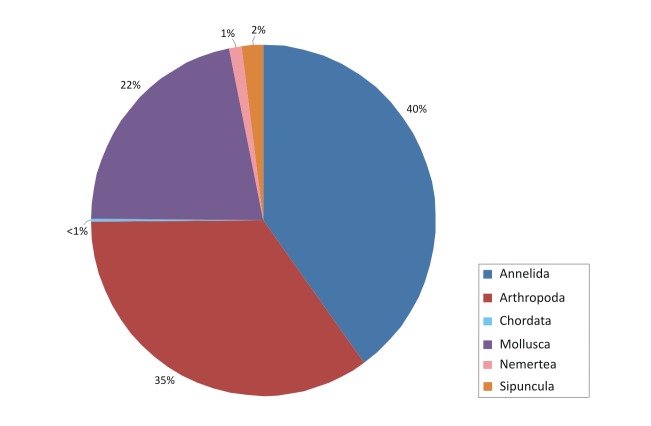
Percentages of main phyla in all the samples of Greece, as calculated based on the individual count of species per total sponge volume.

The contribution of the different phyla found in the samples is shown in the figures below (Figs 4, 5, 6, 7, 8, 9).

In Cyprus samples, Arthropoda was the phylum with the higher representation, both in summer (Fig. 4) and winter samples (Fig. 5). This was evident also when all the samples were included in the analysis, independent of season (Fig. 6). Interestingly, the increased abundance of Echinodermata in the summer (Fig. 4) was substantially reduced in the sponges collected during winter (Fig. 5).

However, the macrofaunal assemblage associated with the sponges collected from Greece was different; Arthropoda were still highly abundant, especially in the summer samples (Fig. 7), but other taxa such as Annelida and Mollusca showed also very high abundances and dominated the winter assemblages (Fig. 8). Overall, independently of season, the importance of the latter two phyla differentiated the sponges collected from the two locations (Fig. 9).

**Figure 10.**
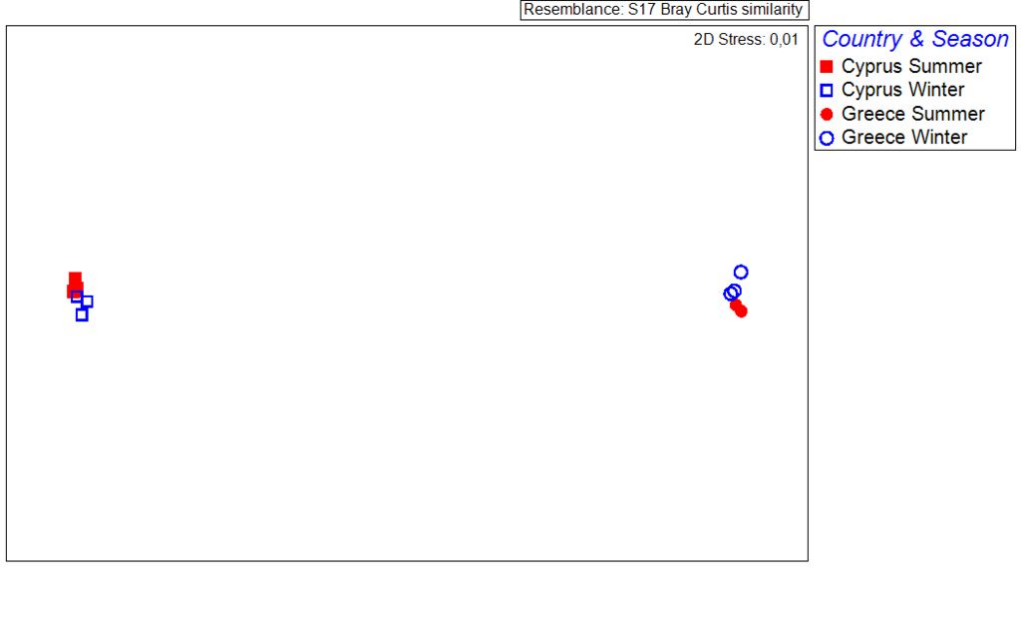
Multidimensional scaling of the sponge samples, based on the individual count of the associated species per sponge volume. Data labels according to the location of the sampling station and the sampling season.

The aforementioned differences in the sponge associated macrofauna are also depicted on the MDS plot (Fig. 10), where it is apparent that the sponge samples cluster per country and season (ANOSIM: R = 0.811, p < 0.01).

**Figure 11.**
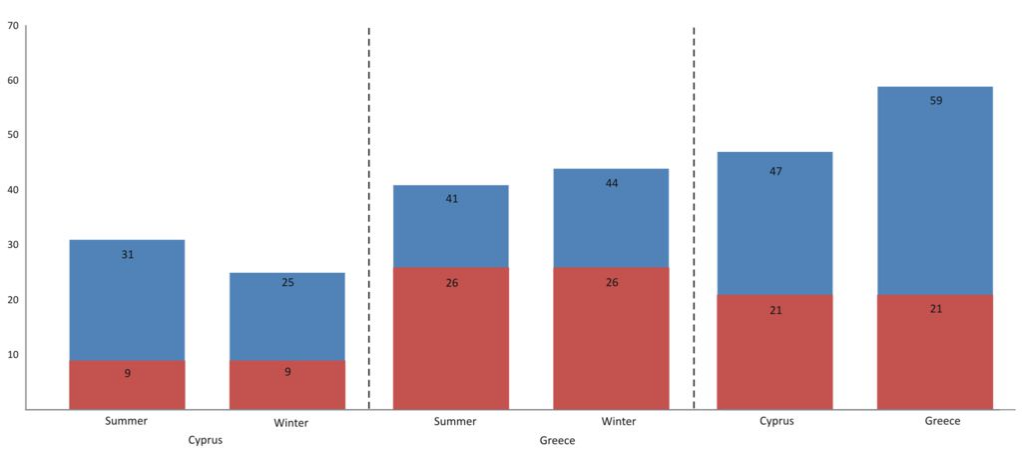
Total number of species (in blue) and common number of species (in red) for both locations and both sampling seasons.

Only 9 macrofaunal species out of the 83 were found to exist in both winter and summer sponge samples from Cyprus. On the contrary, sponges from Greece had 26 species in common when the two seasons were compared. Overall, when the species lists from the sponges collected from Greece and Cyprus were compared, there were 21 species common for both locations (Fig. 11; Table 3).

## Temporal coverage

### Notes

2003-01-01 / 2003-02-282003-07-01 / 2003-08-312007-08-01 / 2007-08-31

## Usage rights

### Use license

Creative Commons CCZero

## Data resources

### Data package title

Macrofaunal assemblages associated with the sponge Sarcotragus foetidus Schmidt, 1862 (Porifera: Demospongiae) at the coasts of Cyprus and Greece

### Resource link

http://lifewww-00.her.hcmr.gr:8080/medobis/resource.do?r=organismic_assemblages_sarcotragus_foetidus_cyprus_greece

### Number of data sets

1

### Data set 1.

#### Data set name

Macrofaunal assemblages associated with the sponge Sarcotragus foetidus Schmidt, 1862 (Porifera: Demospongiae) at the coasts of Cyprus and Greece

#### Data format

Darwin Core Archive

#### Number of columns

33

#### Character set

UTF-8

#### Download URL

http://lifewww-00.her.hcmr.gr:8080/medobis/resource.do?r=organismic_assemblages_sarcotragus_foetidus_cyprus_greece

#### Description

The dataset is available via the MedOBIS (Mediterranean node of Ocean Biogeographic Information System) Internet Publishing Toolkit (IPT) of the Hellenic Centre for Marine Research (HCMR). The data will also be harvested by and made available through the European node of the Ocean Biogeographic Information System (EurOBIS), as well as through the International OBIS database. The dataset is available as a DarwinCoreArchive, all fields are mapped to DarwinCore terms.

This publication refers to the most recent version of the dataset available through the IPT server or MedOBIS. Future changes to the dataset due to quality control activities might change its content or structure.

**Data set 1.** 

Column label	Column description
recordNumber	A unique identifier for the record within the data set or collection
institutionCode	The name (or acronym) in use by the institution having custody of the object(s) or information referred to in the record
basisOfRecord	The specific nature of the data record, as described in http://terms.tdwg.org/wiki/dwc:basisOfRecord
individualCount	The number of individuals in a replicate sample unit
year	The sampling year
month	The sampling month
sampletrackcode	Denotes the code of each sample
fieldNumber	Denotes the code of each replicate unit
continent	The name of the continent in which the sampling location occurs
country	The name of the country in which the sampling location occurs
countryCode	The standard code of the country in which the sampling location occurs
locality	The specific location where the sample was taken
waterBody	The name of the water body in which the sampling location occurs
higherGeographyID	The id of the higher geography of the sampling location according to marineregions.org
minimumDepthInMeters	The lesser depth of a range of depth below the local surface, in meters
maximumDepthInMeters	The greater depth of a range of depth below the local surface, in meters
decimalLatitude	The geographic latitude (in decimal degrees, using the spatial reference system given in geodeticDatum) of the geographic center of a Location. Positive values are north of the Equator, negative values are south of it. Legal values lie between -90 and 90, inclusive
decimalLongitude	The geographic longitude (in decimal degrees, using the spatial reference system given in geodeticDatum) of the geographic center of a Location. Positive values are east of the Greenwich Meridian, negative values are west of it. Legal values lie between -180 and 180, inclusive
coordinateUncertaintyInMeters	The horizontal distance (in meters) from the given decimalLatitude and decimalLongitude describing the smallest circle containing the whole of the sampling location
samplingProtocol	The description of the method or protocol used for sample collection
taxonNameAsInFile	The scientific name of the taxon, as given by the data provider
scientificNameID	A unique identifier for each scientific name
scientificName	The accepted scientific name of the taxon, not including authorship
kingdom	The full scientific name of the kingdom in which the taxon is classified
phylum	The full scientific name of the phylum in which the taxon is classified
class	The full scientific name of the class in which the taxon is classified
order	The full scientific name of the order in which the taxon is classified
family	The full scientific name of the family in which the taxon is classified
genus	The full scientific name of the genus in which the taxon is classified
subgenus	The full scientific name of the subgenus in which the taxon is classified
specificEpithet	The species epithet of the scientificName
scientificNameAuthorship	The authorship information for the scientificName formatted according to the conventions of the applicable nomenclaturalCode
taxonID	Aphia ID for the accepted scientific names (Unique Identifier for the taxon within the World Register of Marine Species - www.marinespecies.org)

## Figures and Tables

**Figure 3a.**
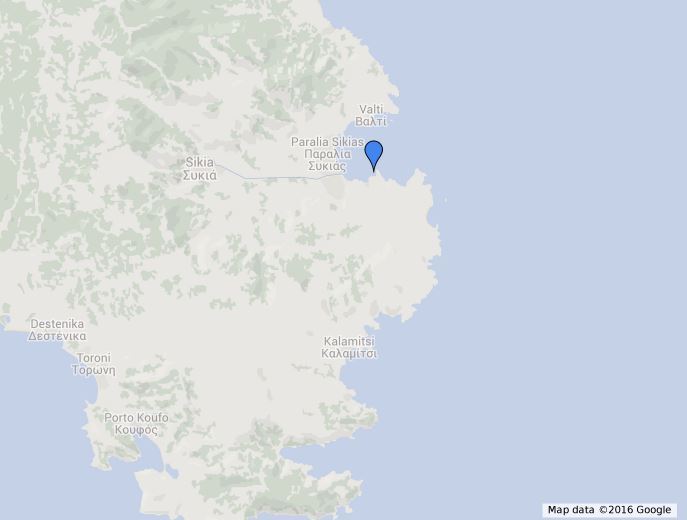
Greece.

**Figure 3b.**
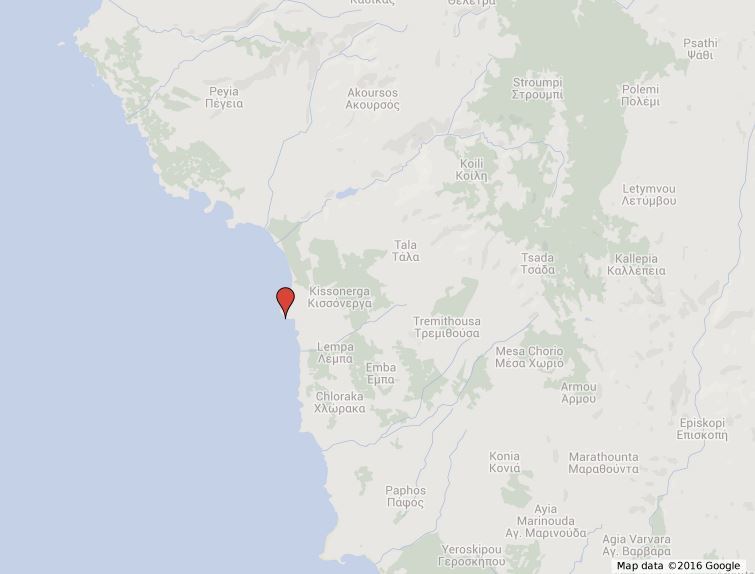
Cyprus.

**Table 1.** Locality, geographical coordinates, depth (m) and physical characteristics of the sampling stations.

Locality	Coordinates	Depth (m)	Habitat
Linaraki Beach, Sykia, Chalkikidi, Greece	40° 2′ 15.003" N 23° 59′ 57.264" E	14 - 17	Rocky shore, moderately exposed. Photophilic algal assemblage dominated by *Ellisolandia elongata* (J.Ellis & Solander) K.R.Hind & G.W.Saunders, 2013
Synthiana’s bay, Milouria, Kissonerga, Paphos, Cyprus	34°′ 18.8148" N 32° 23′ 18.8808" E	5 - 10	Rocky shore, exposed. Photophilic algal assemblage dominated by *Palisada perforata* (Bory de Saint-Vincent) K.W. Nam, 2007

**Table 2.** Taxa identified to the lowest taxonomic level possible and included in the dataset. *: non-indigenous species (ÖZTOPRAK et al. 2014, Pancucci-Papadopoulou et al. 2005). **: identification questionable for individuals from Cyprus (may be in fact Phascolosoma (Phascolosoma) stephensoni) (Açik 2014).

Phylum	Class	Scientific Name	New record for the Levantine Basin
Mollusca	Polyplacophora	*Acanthochitona fascicularis*	
Arthropoda	Malacostraca	*Alpheus dentipes*	
Annelida	Polychaeta	*Amphitrite rubra*	
Annelida	Polychaeta	*Amphitrite variabilis*	
Annelida	Polychaeta	*Arabella iricolor*	
Sipuncula	Phascolosomatidea	Aspidosiphon (Aspidosiphon) muelleri muelleri	
Arthropoda	Malacostraca	*Athanas nitescens*	
Mollusca	Gastropoda	*Bittium reticulatum*	
Annelida	Polychaeta	*Branchiomma bombyx*	
Annelida	Polychaeta	*Branchiosyllis exilis*	
Porifera	Calcarea	Calcarea	
Mollusca	Polyplacophora	*Callochiton septemvalvis*	
Annelida	Polychaeta	*Capitella capitata*	
Arthropoda	Malacostraca	Ceradocus (Ceradocus) orchestiipes	+
Annelida	Polychaeta	Ceratonereis (Composetia) costae	
Annelida	Polychaeta	Ceratonereis (Composetia) hircinicola	
Arthropoda	Malacostraca	*Cestopagurus timidus*	
Arthropoda	Malacostraca	*Colomastix pusilla*	
Mollusca	Gastropoda	*Columbella rustica*	
Arthropoda	Malacostraca	*Cymodoce spinosa*	+
Arthropoda	Malacostraca	*Dexamine spinosa*	
Annelida	Polychaeta	*Dipolydora armata*	+
Annelida	Polychaeta	*Dorvillea rubrovittata*	
Arthropoda	Malacostraca	*Elasmopus pocillimanus*	
Annelida	Polychaeta	*Eunice vittata*	
Arthropoda	Malacostraca	*Eurydice affinis*	+
Arthropoda	Malacostraca	*Galathea intermedia*	
Arthropoda	Malacostraca	*Gammaropsis crenulata*	
Annelida	Polychaeta	*Glycera tesselata*	
Chordata	Actinopteri	*Gobius geniporus*	
Annelida	Polychaeta	*Harmothoe spinifera*	
Mollusca	Bivalvia	*Hiatella arctica*	
Arthropoda	Malacostraca	*Hippolyte leptocerus*	
Annelida	Polychaeta	*Hydroides niger*	
Annelida	Polychaeta	*Hydroides pseudouncinatus*	
Arthropoda	Malacostraca	*Janira maculosa*	+
Annelida	Polychaeta	*Lepidasthenia elegans*	
Arthropoda	Malacostraca	*Leucothoe spinicarpa*	
Arthropoda	Malacostraca	*Liljeborgia dellavallei*	
Mollusca	Bivalvia	*Lima lima*	
Mollusca	Bivalvia	*Lithophaga lithophaga*	
Annelida	Polychaeta	*Lumbrineris coccinea*	
Annelida	Polychaeta	*Lumbrineris latreilli*	
Annelida	Polychaeta	*Lysidice collaris*	
Annelida	Polychaeta	*Lysidice ninetta*	
Arthropoda	Malacostraca	*Lysmata seticaudata*	
Annelida	Polychaeta	*Marphysa sanguinea*	
Arthropoda	Malacostraca	*Microdeutopus bifidus*	
Mollusca	Gastropoda	*Mitra cornicula*	
Nemertea	Nemertea	Nemertea spp.	
Annelida	Polychaeta	*Nereis pelagica*	
Mollusca	Gastropoda	*Ocinebrina aciculata*	
Echinodermata	Ophiuroidea	Ophiactis savignyi*	
Arthropoda	Malacostraca	*Pagurus anachoretus*	
Annelida	Polychaeta	*Palola siciliensis*	
Arthropoda	Malacostraca	*Paractaea monodi*	
Arthropoda	Malacostraca	*Paradoxapseudes intermedius*	+
Annelida	Polychaeta	*Perinereis cultrifera*	
Sipuncula	Phascolosomatidea	Phascolosoma (Phascolosoma) granulatum**	
Annelida	Polychaeta	*Pholoe minuta*	+
Arthropoda	Malacostraca	*Pilumnus spinifer*	
Annelida	Polychaeta	*Platynereis dumerilii*	
Annelida	Polychaeta	*Pontogenia chrysocoma*	
Annelida	Polychaeta	*Psamathe fusca*	
Annelida	Polychaeta	*Pseudopotamilla reniformis*	
Arthropoda	Malacostraca	*Quadrimaera inaequipes*	
Annelida	Polychaeta	*Serpula concharum*	
Annelida	Polychaeta	*Serpula vermicularis*	
Annelida	Polychaeta	*Spirobranchus polytrema*	
Annelida	Polychaeta	*Spirobranchus triqueter*	
Mollusca	Bivalvia	*Striarca lactea*	
Annelida	Polychaeta	*Subadyte pellucida*	
Annelida	Polychaeta	*Syllis armillaris*	
Annelida	Polychaeta	*Syllis columbretensis*	
Annelida	Polychaeta	*Syllis gerlachi*	
Annelida	Polychaeta	*Syllis gracilis*	
Annelida	Polychaeta	*Syllis hyalina*	
Annelida	Polychaeta	*Syllis krohni*	
Annelida	Polychaeta	*Syllis variegata*	
Arthropoda	Malacostraca	*Synalpheus gambarelloides*	
Arthropoda	Malacostraca	*Tanais dulongii*	+
Annelida	Polychaeta	*Trypanosyllis zebra*	
Annelida	Polychaeta	*Vermiliopsis infundibulum*	
Annelida	Polychaeta	*Vermiliopsis monodiscus*	
Annelida	Polychaeta	*Vermiliopsis striaticeps*	

**Table 3.** Species found in common between the samples. *: non-indigenous species (ÖZTOPRAK et al. 2014, Pancucci-Papadopoulou et al. 2005). **: identification questionable for individuals from Cyprus (may be in fact Phascolosoma (Phascolosoma) stephensoni) (Açik 2014).

**Cyprus**	**Greece**	**Cyprus - Greece**
**Summer - Winter**	**Summer - Winter**	
*Cestopagurus timidus*	*Alpheus dentipes*	*Alpheus dentipes*
*Cymodoce spinosa*	*Amphitrite variabilis*	Aspidosiphon (Aspidosiphon) muelleri muelleri
*Elasmopus pocillimanus*	*Athanas nitescens*	*Bittium reticulatum*
*Leucothoe spinicarpa*	*Bittium reticulatum*	*Cestopagurus timidus*
*Microdeutopus bifidus*	Ceratonereis (Composetia) costae	*Colomastix pusilla*
Ophiactis savignyi*	Ceratonereis (Composetia) hircinicola	*Dipolydora armata*
Phascolosoma (Phascolosoma) granulatum**	*Cestopagurus timidus*	*Janira maculosa*
*Quadrimaera inaequipes*	*Colomastix pusilla*	*Leucothoe spinicarpa*
*Synalpheus gambarelloides*	*Harmothoe spinifera*	*Liljeborgia dellavallei*
	*Hiatella arctica*	*Lysidice collaris*
	*Hippolyte leptocerus*	Nemertea spp.
	*Janira maculosa*	*Nereis pelagica*
	*Lepidasthenia elegans*	*Palola siciliensis*
	*Leucothoe spinicarpa*	*Paradoxapseudes intermedius*
	*Liljeborgia dellavallei*	Phascolosoma (Phascolosoma) granulatum**
	*Lumbrineris coccinea*	*Pseudopotamilla reniformis*
	*Lumbrineris latreilli*	*Quadrimaera inaequipes*
	*Lysidice collaris*	*Serpula vermicularis*
	Nemertea spp.	*Striarca lactea*
	*Palola siciliensis*	*Synalpheus gambarelloides*
	Phascolosoma (Phascolosoma) granulatum	*Vermiliopsis striaticeps*
	*Spirobranchus triqueter*	
	*Subadyte pellucida*	
	*Syllis gracilis*	
	*Synalpheus gambarelloides*	
	*Vermiliopsis monodiscus*	
